# Online Medical Literature Consultation Habits of Academic Teaching Physicians in the EU and CIS Countries: A Cross-Sectional Study

**DOI:** 10.1371/journal.pone.0044302

**Published:** 2012-11-02

**Authors:** Chiel T. M. van der Voort, Cees A. Swenne, Catharina A. M. van der Hoorn-van Velthoven, Johannes H. J. Belt

**Affiliations:** 1 Walaeus Library, Leiden University Medical Center, Leiden, The Netherlands; 2 Department of Cardiology, Leiden University Medical Center, Leiden, The Netherlands; The Cochrane Collaboration, Germany

## Abstract

**Background:**

Both in the Commonwealth of Independent States (CIS) and in the European Union (EU2004), ample availability of up to date medical scientific literature is important for progress in medical science and for the education of the next generation of healthcare workers. The aim of this research is to assess if the use of online medical literature among academic teaching (AT) physicians is at the same level in the CIS as in the EU2004.

**Methodology/Principal Findings:**

In the capital cities of the CIS and the EU2004 member states, AT physicians holding an academic position at least equivalent to an associate professor and performing the three classical tasks in academic medicine (teaching, research and patient care) were interviewed about their use of and familiarity with the Internet and 9 online literature services, including journals and bibliographical databases such as PubMed (Medline), The Cochrane Library and Web of Science. Library staff members were interviewed about the availability of these online literature services at their libraries. About 750 physicians and 40 library staff members were invited for participation. Eventually 124 AT physicians and 22 library staff members participated. Internet was everywhere available, but used daily by more AT physicians in the EU2004 (71% versus 48% in the CIS, *P* = .005). AT physicians in the EU2004 accessed a higher percentage of all articles online (74% versus 43% in the CIS, *P*<.001). PubMed (*P*<.001), The Cochrane Library (*P*<.001) and Web of Science (*P*<.003) were used more frequently in the EU2004. In the EU2004 more AT physicians were familiar with Open Access journals (89% versus 51% in the CIS, *P*<.001).

**Conclusions/Significance:**

AT physicians in the CIS use online medical literature less than in the EU2004. It is recommended that the awareness of freely available online literature services such as Open Access journals is enhanced among AT physicians and library staff members, especially in the CIS.

## Introduction

The importance of up to date medical scientific literature to improve healthcare, ensure progress in medical sciences and educate the next generation of healthcare workers, has long been established [1]–[8]. It can be said that up to date medical scientific literature should be available to every individual working in the healthcare sector of any country. The Internet, in combination with the digitization of medical scientific literature and the surge of Open Access journals, facilitates this goal greatly [9].

In the early 90's of the last century hardly any online medical literature was used in the Western world. Academic teaching (AT) physicians mainly relied on textbooks, hard copy journals and colleagues as sources of medical information [1]–[4], [10]–[12]. During the second half of the 90's the number of AT physicians using online medical literature rose [12]–[19]. The pace of this transition increased tremendously throughout the first decade of the 21^st^ century [20]–[29]. Nowadays, online medical literature is used at a large scale within the Western world, including the European Union (in the following abbreviated as EU2004, which stands for all member states who joined before May 2004, excluding Luxembourg: Austria, Belgium, Denmark, Finland, France, Germany, Greece, Ireland, Italy, Portugal, Spain, Sweden, The Netherlands and United Kingdom) [29], [30]. In the last 15 years medical libraries in the EU2004 evolved in concordance with the digitization and replaced many of their hard copy journals with online journals [25], [26], [31], [32].

While the Internet facilities of healthcare systems within the member states of the EU2004 developed, the Commonwealth of Independent States (in the following abbreviated as CIS; comprising Armenia, Azerbaijan, Belarus, Kazakhstan, Kyrgyzstan, Moldova, Russia, Tajikistan, Ukraine and Uzbekistan) had to deal with the consequences of the partitioning of the Soviet Union. All member states experienced severe economical hardship, which resulted in enormous difficulties in many aspects. In the sphere of medicine these were exaggerated by the oversized and inefficient healthcare systems which the newly independent states inherited from the Soviet Union [33]–[47]. The effect on research is indicated by a lower amount of publications in the CIS [48]–[53]. With far reaching financial constraints in mind, it is reasonable to presume that institutions in the CIS did not improve their Internet facilities at the same rate as in the EU2004. This is indicated by a consistently lower percentage of internet users in CIS countries as opposed to EU2004 countries [54]. Hence the availability of online medical literature is likely to be less. Because of this and an assumed lower proficiency in the English language, one could expect that the use of online medical literature among AT physicians in the CIS is not as integrated into daily practise yet, as among AT physicians in the EU2004. This study assesses if the use and availability of online medical literature among AT physicians in the CIS is at the same level as in the EU2004.

## Methods

### Ethics Statement

As our study does not regard medical research on humans or animals, but rather is concerned with the logistics of medical literature consultation, it is not subject to the Declaration of Helsinki, and no approval by a medical ethics committee is required.

### Sample

We aimed to interview at least five AT physicians in every participating capital city of both the 14 EU2004 and 10 CIS countries. AT physicians were considered eligible for participation if they 1) held an academic position at least equivalent to an associate professor and if they 2) performed the three classical tasks in academic medicine: teaching, research and patient care [55]. In order to be qualified as a researcher they had to be the author of a minimum of 20 scientific publications as well as take part in peer reviewing of medical scientific literature. We focused on this specific subgroup of AT physicians because these are the academic professors training the next generation of medical professionals. Therefore they are likely to have most impact on the future of medical practise in their countries.

### Recruitment

From April 2009 till February 2010 the first author visited hospitals, medical faculties and medical libraries in all 24 included capital cities (Amsterdam, Astana, Athens, Baku, Berlin, Bishkek, Brussels, Chisinau, Copenhagen, Dublin, Dushanbe, Helsinki, Kiev, Lisbon, London, Madrid, Minsk, Moscow, Paris, Rome, Stockholm, Tashkent, Vienna and Yerevan) (Table 1). In order to enroll participants the interviewer tried to recruit a contact person in a medical library or at a medical faculty dean's office by e-mail prior to the visit. University hospital websites were scanned for e-mail addresses of AT physicians. These AT physicians were asked if they were or knew an eligible physician who would be willing to participate. If, in spite of these efforts, an insufficient amount of participants was recruited, the first author consulted deans or rectors at the medical faculty, personnel from medical libraries and actual participants for other potential participants during his visit. If this did not result in sufficient participants, more AT physicians were contacted post-hoc by e-mail. The group characteristics of participating AT physicians and librarians are listed in table 2 and 3 respectively. In the capital cities where all participating AT physicians worked at the same institution the library of that institution was selected, unless participants mentioned that another library was more important to them. Whenever participating AT physicians were located at different institutions, one or more libraries were selected based on directions from the contact person or participating AT physicians in that capital. Eventually per capital the most comprehensive library was used for analysis.

**Table 1:** Participating institutions and libraries.

City	Institution	Participants	Library
Amsterdam	Amsterdam Medical Center	5	Amsterdam Medical Center
Astana	Medical University Astana	7	Medical University Astana
Athens	Several	6	University of Athens
Baku	State Medical University	8	State Medical University
Berlin	Charité	5	Charité
Bishkek	Kyrgyz State Medical Institute	5	Kyrgyz State Medical Institute
Brussels	University Hospital Brussels	5	University Hospital Brussels
Chisinau	State Medical and Pharmaceutical University	5	State Medical and Pharmaceutical University
Copenhagen	Rigshospitalet	5	Royal Library
Dublin	Several	5	Trinitiy's college
Dushanbe	Tajik State Medical Institute	5	Tajik State Medical Institute
Helsinki	Helsinki University Central Hospital	5	National Library of Health Sciences
Kiev	Several	6	National Scientific Medical Library Ukraine
Lisbon	Medical Faculty of Lisbon	6	Medical Faculty of Lisbon
London	St George's University of London	5	St George's University of London
Madrid	Clinical Hospital San Carlos	6	Clinical Hospital San Carlos
Moscow	Several	5	Russian State Medical University
Rome	Catholic University of the Holy Heart of Rome	5	Catholic University of the Holy Heart of Rome
Stockholm	Karolinska Institutet	5	Karolinska Institutet
Tashkent	Tashkent State Medical Institute	6	Scientific Medical State Library
Vienna	Medical University Vienna	7	Medical University Vienna
Yerevan	Yerevan Medical State University	7	Yerevan Medical State University

**Table 2:** Group characteristics of participating AT physicians in the EU2004 and the CIS.

	EU2004	CIS	*P*-value
N	70	54	
Sex, male (%)	61 (87)	43 (80)	.26
Mean age	54	51	.16
Reads English (%)	70 (100)	44 (82)	<.001
Speaks English (%)	70 (100)	34 (63)	<.001
Reads Russian (%)	1 (1)	54 (100)	<.001
Speaks Russian (%)	0 (0)	54 (100)	<.001
Face to face^a^ (%)	59 (84)	41 (76)	.24
Number of articles read per week	7.9	6.7	.11

a data was acquired face to face as opposed to by correspondence.

**Table 3:** Group characteristics of participating library staff members in the EU2004 and the CIS.

	EU2004	CIS	*P*-value
N	13	9	
Sex, male (%)	4 (31)	0 (0)	.07
Mean age^a^	47	49	.71
University degree (%)	70 (100)	9 (100)	NA
Face to face^b^ (%)	12 (92)	7 (78)	.34

a missing EU2004 N = 1, CIS N = 3.

b data was acquired face to face as opposed to by correspondence.

### Procedure

A questionnaire (in the English or Russian language, whichever the participant preferred) containing 42 questions was presented to every participating physician (see Appendix 1). Answers to the questionnaires were obtained face to face or by correspondence, if the contact was made post-hoc. The questionnaires consisted of a mix of open and closed questions. Questions regarded the languages the participant was able to read and speak, the availability, use and speed of the Internet, the experience with the Internet, the use of online journals, the most frequently read online and hardcopy journals, the percentage of scientific articles read online, the familiarity with the concept of Open Access journals and the familiarity with and the use of certain online literature services, including bibliographical databases and journals (PubMed (Medline), Embase, Web of Science, The Cochrane Library, Public Library of Science (PLoS), BioMedCentral, British Medical Journal, Medscape, and HINARI Programme for Access to Health Research (HINARI)). We focused on these literature services because of their medically relevant content. HINARI was especially included because most CIS countries are eligible to free or affordable access. Participants was given the opportunity to mention others to avoid overlooking relevant online literature services beyond the above mentioned.

As there can be a discrepancy between the actual use and the availability of online literature services, an English or Russian questionnaire was presented to the staff of medical libraries as well (see Appendix 2). This questionnaire contained 38 questions concerning the library's staff members' level of education, the familiarity with and the availability of certain online literature services and their preferences for online literature services.

### Statistical analysis

The AT physicians were compared according to the following factors: demographic characteristics, ability to read and speak English and Russian, whether the interview was conducted face to face or by correspondence, the percentage of articles accessed online, how often the participant used online journals, whether the participant was familiar with Open Access journals, where and how often the participant accessed the Internet, how the participant qualified the speed of the Internet, whether the participant considered himself experienced with personal computers and the Internet and whether the participant was familiar with and how often the participant accessed certain online literature services.

The library staff members were compared according to the following factors: demographic characteristics, whether the interview was conducted face to face or by correspondence and which online literature services were available at their institutions.

To compare the EU2004 with the CIS results, chi-square-tests and independent t-tests were conducted. If in a chi-square-test the cell count was less than 5 in at least 20% of the cells, a Mann-Whitney-test was conducted instead. A *P*-value less than .05 was considered significant.

## Results

### Demographic characteristics

Of the about 750 physicians and 40 library staff members who were initially invited, 124 AT physicians and 22 library staff members participated in our study (Table 1, 2 and 3). The aim to include at least five AT physicians per capital city was attained in 22 out of the 24 capitals. In Minsk and Paris the minimum requirements were not met, since insufficient AT physicians were available for participation during the first author's visit, and too few AT physicians responded to the request for participation by e-mail. As a consequence, no contributions from Minsk and Paris were incorporated in this study.

### The Internet

**Figure 1:**
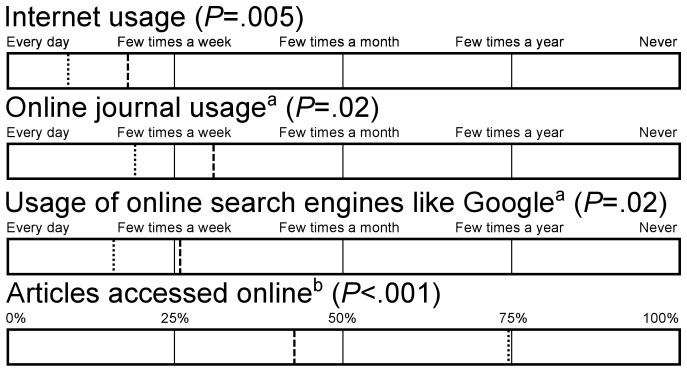
Usage of Internet and information resources by AT physicians in EU2004 and the CIS. Dotted line = EU2004, dashed line = CIS. EU2004 N = 70, CIS N = 54. ^a^ missing CIS N = 2. ^b^ percentage of articles accessed online as part of the articles read, missing CIS N = 1.

All AT physicians (100%) had access to the Internet. More AT physicians in the EU2004 (71%) than their counterparts in the CIS (48%) used the Internet on a daily basis (*P* = .005) and reported to have their own computer at work (96% versus 63%, *P*<.001). The speed of the Internet was considered to be fast by more AT physicians in the EU2004 (84%) than in the CIS (50%, *P*<.001). AT physicians in the EU2004 turned out to have more experience with the Internet than their colleagues in the CIS (*P*<.001). In the EU2004 93% of the AT physicians answered to ‘definitely’ have experience with the Internet, while in the CIS 69% did so (Table 4). AT physicians in the EU2004 used online search engines like Google more often than their colleagues in the CIS (*P* = .02) (Figure 1).

**Table 4:** Daily Internet usage, main location of Internet, speed of Internet, experience with personal computers, Internet and online literature services among AT physicians in EU2004 and the CIS.

	EU2004	CIS	*P*-value
N	70	54	
General Internet usage			.005
Every day (%)	50 (71)	26 (48)	
Main location of computer			<.001
Own computer at work (%)	67 (96)	34 (63)	
At home (%)	1 (1)	15 (28)	
Shared computer at work (%)	2 (3)	4 (7)	
Library (%)	0 (0)	1 (2)	
Speed of Internet connection			<.001
Fast (%)	60 (84)	27 (50)	
Slow but workable (%)	11 (16)	22 (41)	
Too slow (%)	0 (0)	5 (9)	
Experience with computers			.35
Definitely (%)	57 (81)	40 (74)	
Some experience (%)	11 (16)	14 (26)	
No experience (%)	1 (1)	0 (0)	
Experience with the Internet			<.001
Definitely (%)	65 (93)	37 (69)	
Some experience (%)	4 (6)	13 (24)	
No experience (%)	1 (1)	4 (7)	
Experience with online literature services			.01
Definitely (%)	63 (90)	39 (72)	
Some experience (%)	6 (9)	12 (22)	
No experience (%)	1 (1)	3 (6)	

### Journal readership

AT physicians in the CIS read on average 6.7 articles per week, while AT physicians in the EU2004 read 7.9 articles per week (P = .11) (Table 2). Online journals were consulted significantly less by AT physicians in the CIS compared with AT physicians in the EU2004 (*P* = .02). The AT physicians in the EU2004 estimated that on average they accessed 74% of all articles online. In the CIS the AT physicians estimated that of the articles read, they accessed 43% online (*P*<.001) (Figure 1). In the EU2004 56 out of 63 AT physicians (89%) were familiar with Open Access journals, in contrast to 22 out of 43 (51%) AT physicians in the CIS (*P*<.001).

**Figure 2:**
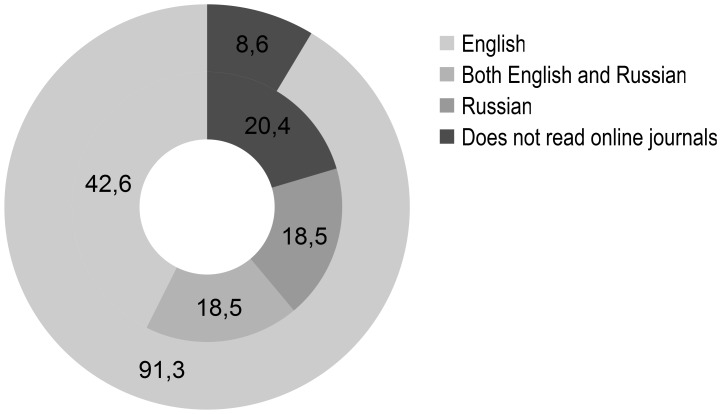
Language of 4 most read online journals among AT physicians (%). Inner circle = CIS (N = 54), outer circle = EU2004 (N = 69).

Participants were asked to list the 4 journals they read online most. In the CIS 11 AT physicians (20%) stated not to read journals online. In the EU2004 6 AT physicians (9%) did not read journals online (*P* = .06). Among the AT physicians who read online journals, in the CIS 23 AT physicians (54%) listed journals published in English only, while 10 (23%) listed both English and Russian journals. Of the CIS AT physicians 10 (23%) exclusively listed journals published in Russian. Of the 63 AT physicians in the EU2004 who read online journals, all (100%) listed journals published in English (Figure 2).

**Figure 3:**
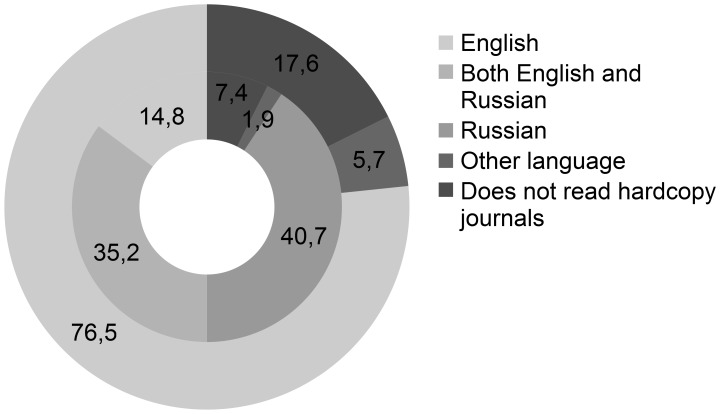
Language of 4 most read hardcopy journals among AT physicians (%). Inner circle = CIS (N = 54), outer circle = EU2004 (N = 69).

In the CIS 4 AT physicians (7%) did not read hardcopy journals. In the EU2004 12 AT physicians (18%) did not (*P* = .1). From the AT physicians who did read hardcopy journals, one physician (2%) in the CIS exclusively read journals in a language other than English or Russian. In the EU2004 4 AT physicians (7%) exclusively read hardcopy journals in a language other than English or Russian. However both in the CIS and in the EU2004 these participants read English journals online. All of the other hardcopy journal reading physician (N = 63, 93%) in the EU2004 read journals published in English. In the CIS 8 (16%) exclusively listed journals published in English, while 19 (38%) mentioned both English and Russian jourals and 22 (44%) solely read Russian hardcopy journals (Figure 3).

### Online literature services

In the EU2004 AT physicians were more familiar with certain online literature services (bibliographical databases and journals) than in the CIS (90% versus 72%, *P* = .01) (Table 4). A higher percentage of AT physicians in the EU2004 than in the CIS was familiar with PubMed, which lists Russian as well as English articles (100% versus 89%, *P* = .004). A higher percentage of AT physicians in the EU2004 was aware of The Cochrane Library (99% versus 57%, *P*<.001), PLoS (69% versus 46%, *P* = .01), Web of Science (77% versus 37%, *P*<.001) and EMBASE (70% versus 24%, *P*<.001) as well.

According to library staff members availability of online literature services widely varied. They were all aware that PubMed is freely accessible on the Internet. HINARI, an initiative of the World Health Organization aiming to make medical scientific journals available to health care workers in countries with a gross national income below 3500$ per capita [56] was only available at 2 out of 8 eligible CIS' libraries (25%). Web of Science was available at all of the 13 libraries in the EU2004 (100%), but at none of the libraries in the CIS (0%) (*P*<.001). The Cochrane Library (92% versus 33%, *P* = .004) and Embase (69% versus 0%, *P* = .002) were more available in the EU2004 as well. Despite it being a freely accessible service, Medscape was acknowledged to be available at 3 (33%) of the CIS' and none (0%) of the EU2004 libraries (*P* = .03) (Table 5 and 6) [57]. British Medical Journal provides free access to its articles to all CIS countries but Russia [58]. In 5 out of 8 (63%) eligible CIS libraries this was acknowledged (Table 5 and 6). PLoS and BioMedCentral, both exclusively providing freely accessible Open Access journals, were acknowledged to be available in 2 out of 9 (22%) libraries in the CIS. In the EU2004 respectively 69% and 77% of librarians acknowledged PLoS and BioMedCentral to be available (Table 5 and 6).

**Table 5:** Availability of free and paid for online literature services in participating institutions.

City where Library is located		PubMed	The Cochrane Library	British Medical Journal	Medscape	PLoS	Web of Science	BioMedCentral	Embase	HINARI
CIS	Astana	X	.	X	.	X	.	.	.	.
	Baku	X	.	.	X	.	.	.	.	**.**
	Bishkek	X	X	.	.	.	.	.	.	.
	Chisinau	X	.	X	X	.	.	X	.	X
	Dushanbe	X	X	X	.	.	.	.	.	.
	Kiev	X	X	X	X	X	.	.	.	**.**
	Moscow	X	.	X	.	.	.	X	.	.
	Tashkent	X	.	X	.	.	.	.	.	.
	Yerevan	X	.	.	.	.	.	.	.	**X**
EU2004	Amsterdam	X	X	X	.	X	X	X	X	.
	Athens	X	X	X	.	.	X	X	.	.
	Berlin	X	X	X	.	X	X	X	X	.
	Brussels	X	.	.	.	.	X	.	.	.
	Copenhagen	X	X	.	.	X	X	X	X	.
	Dublin	X	X	X	.	X	X	X	X	.
	Helsinki	X	X	X	.	X	X	X	X	.
	Lisbon	X	X	X	.	X	X	X	.	.
	London	X	X	X	.	X	X	X	X	.
	Madrid	X	X	X	.	.	X	.	X	.
	Rome	X	X	.	.	.	X	.	.	.
	Stockholm	X	X	X	.	X	X	X	X	.
	Vienna	X	X	X	.	X	X	X	X	.

X: Institution subscribed. Full stop: Institution not subscribed. Underlined: Institution eligible for free access. Bold: Institution eligible to access HINARI for a fee of $1000 per institution per year.

**Table 6:** Percentage of AT physicians in the EU2004 and the CIS familiar with certain online literature services and the availability of online literature services according to library staff members.

	Familiarity among Physicians			Availability in libraries		
	EU2004 (%)	CIS (%)	*P*-value	EU2004 (%)	CIS (%)	*P*-value
N (physicians, library staff members)	70	54		13	9	
PubMed	70 (100)	48 (89)	.004	13 (100)	9 (100)	NA
The Cochrane Library	69 (99)	31 (57)	<.001	12 (92)	3 (33)	.004
British Medical Journal^a^	53 (76)	37 (69)	.37	10 (77)	6 (67)	.60
Medscape	44 (63)	28 (52)	.22	0 (0)	3 (33)	.03
PLoS^b^	48 (69)	25 (46)	.01	9 (69)	2 (22)	.03
Web of Science	54 (77)	20 (37)	<.001	13 (100)	0 (0)	<.001
BioMedCentral	44 (63)	27 (50)	.15	10 (77)	2 (22)	.01
Embase^c^	49 (70)	13 (24)	<.001	9 (69)	0 (0)	.002
HINARI^d^	9 (13)	12 (22)	.17	0 (0)	2 (22)	.08

a the online platform of the British Medical Journal.

b Public Library of Science.

c Excerpta Medica Database.

d World Health Organisation Program for Access to Health Research.

**Figure 4:**
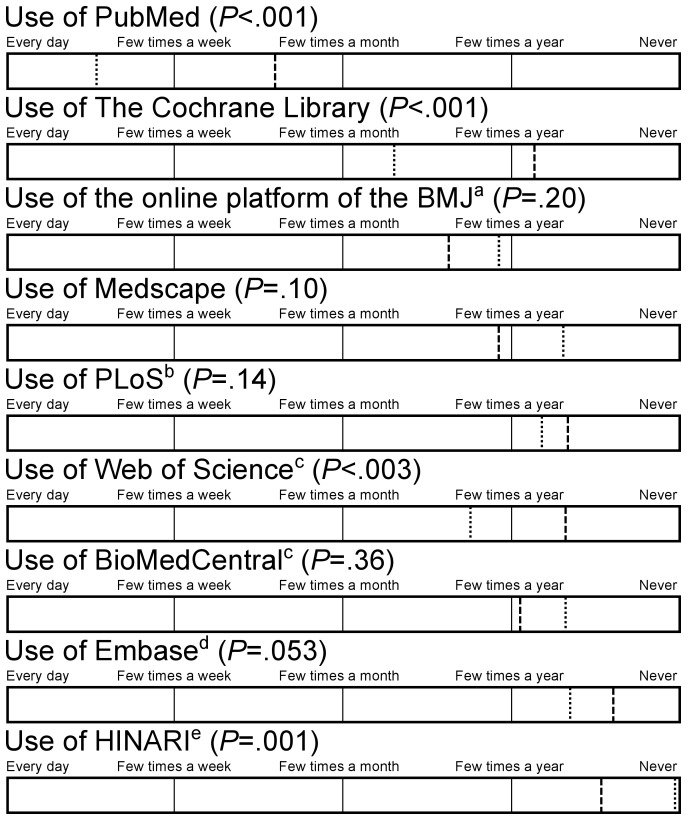
AT physicians' use of online literature services in EU2004 and the CIS. Dotted line = EU2004, dashed line = CIS. EU2004 N = 70, CIS N = 54. ^a^ British Medical Journal, ^b^ Public Library of Science, ^c^ missing CIS N = 1, ^d^ Excerpta Medica Database, ^e^ World Health Organisation Program for Access to Health Research.

The library staff members were asked to choose their favourite literature service out of a list of 9 online literature services. From them 18 out of 22 (83%) preferred PubMed, stating that it was free and easy to use. PubMed (*P*<.001) and The Cochrane Library (*P*<.001) were used more often in the EU2004 than in the CIS. HINARI was the least known online literature service and used hardly ever by AT physicians in the CIS, that would qualify for this service (Figure 4).

To assure that no frequently used online literature service was left out by our questionnaire all AT physicians were asked which services they used besides these 9. No other online literature services turned out to be of significance to the participants. Some Russian services were mentioned, but in all cases they were specialism specific and mentioned by one participant only.

## Discussion

### Main results

Every participant had access to the Internet. However, in the EU2004 the Internet is faster, used more frequently and a higher percentage of articles is accessed online (Figure 1, Table 4). This could be partly ascribed to the fact that in the CIS fewer AT physicians have their own computer at work (Table 4). Moreover, less online literature services are available for AT physicians in the CIS (Table 5 and 6). Hence, AT physicians in the CIS are less familiar with, and use more occasionally, online literature services than their colleagues in the EU2004 (Table 6, Figure 4). This is reflected by a lower readership of online journals among AT physicians in the CIS (Figure 1), despite a similar amount of articles read per week (Table 2). Both in the EU2004 and in the CIS online journals were mostly read in English, although 23% of AT physicians mentioned to read online Russian journals exclusively (Figure 2). The preferred language of hardcopy journals among AT physicians in the CIS was Russian, with only 16% mentioning English language journals exclusively (Figure 3).

### Comparison with prior work

In the EU2004, the most often used online literature services are the databases: PubMed, which is ubiquitously available, The Cochrane Library, available in 12 out of 13 libraries, and Web of Science, which is available in all of the EU2004' libraries (Figure 4, Table 5 and 6). These figures are consistent with existing evidence from Cullen and Hider in New Zealand and Schilling in Colorado, who found PubMed to be the online literature service most often used. Cullen mentioned The Cochrane Library to rank third [20], [29], [59]. In the CIS, PubMed is followed by the online platform of the British Medical Journal, where this publisher made its journal freely available and which is reported to be available in 6 out of 9 medical libraries, and by Medscape, an information portal which is reported to be available in 3 out of 9 CIS' libraries (Figure 4, Table 5 and 6).

The lack of available online literature services in the CIS can partly be accredited to the lack of financial means. This is illustrated by above mentioned top 3's of online literature service usage. The one's which are used in the CIS, namely PubMed, the British Medical Journal and Medscape, can be used for free in these countries. In contrast, apart from PubMed, AT physicians in the EU2004 resort to paid services like the Cochrane Library and Web of Science (Figure 4). Interestingly Medscape was thought to be available only at 3 out of 9 CIS libraries and none of the 13 EU2004 libraries. Apparently institutions in the EU2004 do not pay attention to this free online literature service.

Based on the fact that only 2 (22%) of the libraries in the CIS recognized Open Access journal providers PLoS and BioMedCentral to be accessible, unawareness among library staff members of freely accessible online information sources appears to be an important factor as well. Yet, approximately half of the AT physicians in the CIS were familiar with these 2 services (Table 6). It seems that in some cases AT physicians are able to reach freely accessible online literature services better than library staff members. While the need for freely accessible online literature services is actually more profound in the CIS, AT physicians in the EU2004 are more familiar with the concept of Open Access Journals. In this same aspect it is remarkable that HINARI was only available at 2 out of 8 eligible CIS' libraries and hardly recognized by AT physicians in the CIS (Table 6).

### Limitations

This study only focused on physicians in capital cities who 1) held an academic degree at least equivalent to associate professor, and 2) performed the three classical tasks in academic medicine: teaching, research and patient care. Hence this study can not be extrapolated to the majority of physicians practising in the CIS and the EU2004. The fact that only AT physicians working at institutions in capital cities were invited for participation may have influenced our results. It may be presumed that in the CIS funds are more likely to be invested in the capital cities [33]–[47]. Therefore the situation as outlined here, might be the best case scenario for many of the participating members of the CIS. As the participating physicians in the academic hospitals are teaching the next generation of physicians they are a very relevant group to focus on.

The results of this research project may have been influenced by several confounders. More active or better English speaking AT physicians may have been recommended by their colleagues, library staff members and deans more often, and may have been more willing to participate. AT physicians with a more active attitude or who have better English skills are more likely to use online literature services more frequently. This could have caused a selection bias. However, more active AT physicians may have had a tight time-schedule and therefore might have been less willing to participate. Participants may have been inclined to give more favourable answers, which could have influenced the outcome. The questionnaires were not validated. However, we have no reason to assume that the above mentioned confounders influenced a particular region more than the other.

### Conclusions

The use of online medical literature (bibliographic databases, full text) is lower among AT physicians in the CIS compared to AT physicians in the EU2004. AT physicians in the EU2004 access a higher percentage of articles online, they use the Internet more frequently and they are more familiar with certain online literature services, including freely accessible ones such as Open Access journals. It is recommended that in the CIS internet facilities within medical institutions are improved, as well as the awareness and provision of services available at the medical libraries. Particularly awareness of freely available online bibliographic databases should be enhanced, especially, and in the case of HINARI exclusively, among AT physicians and library staff members in the CIS.
